# Study on the Overmolding Process of Carbon-Fiber-Reinforced Poly (Aryl Ether Ketone) (PAEK)/Poly (Ether Ether Ketone) (PEEK) Thermoplastic Composites

**DOI:** 10.3390/ma16124456

**Published:** 2023-06-18

**Authors:** Ziyue Zhao, Jindong Zhang, Ran Bi, Chunhai Chen, Jianan Yao, Gang Liu

**Affiliations:** Center for Advanced Low-Dimension Materials, State Key Laboratory for Modification of Chemical Fibers and Polymer Materials, College of Materials Science and Engineering, Donghua University, Shanghai 201620, China

**Keywords:** poly (aryl ether ketone), poly (ether ether ketone), overmolding, interfacial properties, molecular dynamics simulation

## Abstract

This paper used poly (aryl ether ketone) (PAEK) resin with a low melting temperature to prepare laminate via the compression-molding process for continuous-carbon-fiber-reinforced composites (CCF-PAEK). Then, poly (ether ether ketone) (PEEK), or a short-carbon-fiber-reinforced poly (ether ether ketone) (SCF-PEEK) with a high melting temperature, was injected to prepare the overmolding composites. The shear strength of short beams was used to characterize the interface bonding strength of composites. The results showed that the interface properties of the composite were affected by the interface temperature, which was adjusted by mold temperature. PAEK and PEEK formed a better interfacial bonding at higher interface temperatures. The shear strength of the SCF-PEEK/CCF-PAEK short beam was 77 MPa when the mold temperature was 220 °C and 85 MPa when the mold temperature was raised to 260 °C. The melting temperature did not significantly affect the shear strength of SCF-PEEK/CCF-PAEK short beams. For the melting temperature increasing from 380 °C to 420 °C, the shear strength of the SCF-PEEK/CCF-PAEK short beam ranged from 83 MPa to 87 MPa. The microstructure and failure morphology of the composite was observed using an optical microscope. A molecular dynamics model was established to simulate the adhesion of PAEK and PEEK at different mold temperatures. The interfacial bonding energy and diffusion coefficient agreed with the experimental results.

## 1. Introduction

High-performance thermoplastic composites have advantages such as high toughness, impact resistance, low moisture absorption rate, and excellent environmental resistance. Its raw materials can be transported and stored at room temperature. Moreover, it shows a fast-forming and secondary-forming speed [1,2,3]. Good welding, recycling, and reprocessing are also among their characteristics, which is why it has been widely used in aerospace technologies [4,5]. High-performance thermoplastic composites have advantages in fabricating cabin doors, covers, and other parts vulnerable to impact damage and must be loaded and unloaded repeatedly. It can effectively compensate for the inherent problems of insufficient interlaminar toughness, poor impact damage resistance, and poor open-hole strength of thermosetting composites. Typical high-performance thermoplastic composites include poly (ether ether ketone) (PEEK) [6], poly (aryl ether ketone) (PAEK), poly (phenylene sulfide) (PPS), poly (ether imide) (PEI), and other resin-based composites [7,8,9]. Its primary forming process includes molding, autoclave forming, automatic laying forming, welding forming, and stamping forming processes [10]. Compared with the forming process of thermosetting composites, thermoplastic composites have a short and flexible forming cycle. For locally reinforced structures, such as stiffened siding, thermosetting composites are usually co-cured, glued, or riveted [11]. However, thermoplastic composites are generally formed by hot pressing. Then the reinforced panel structure is manufactured by unique connection processes of thermoplastic composites, such as induction and resistance welding [12,13,14]. Although the molding cycle is shortened to a certain extent, the welding process needs special equipment, and the secondary molding will lead to higher manufacturing costs. Therefore, the automotive industry commonly uses the overmolding [15,16,17,18] process of thermoplastic composites. Studying the overmolding process of high-performance thermoplastic composites for aerospace applications is of great engineering significance.

The overmolding process refers to the combination of the bearing efficiency of the continuous-fiber-reinforced thermoplastic composite structure and the manufacturing flexibility of the injection molding process of the short-carbon-fiber-reinforced thermoplastic composite. This is carried out to realize the low-cost integrated manufacturing of thermoplastic composites. Many studies have shown that the interfacial bonding of bilayer structures mainly depends on mechanical locking and resin-fusion mechanisms, and the latter is the main influencing factor. Aurrekoetxea et al. [19] studied the influence of the processing conditions on the interfacial bonding strength when polypropylene (PP) was injected into PP laminate. The interfacial bonding strength was superior when the temperature exceeded the matrix resin melting point. Robert Boros et al. [20] studied the influence of plasma treatment on the interfacial bonding strength of overmolding. The results show that the bonding strength of ABS/PA6 can reach 12 MPa after plasma treatment, but there is no bonding when untreated. Koki Matsumo et al. [21] studied a new method to realize the nanoscale interconnection between different layers by inserting thin films between them. The results show that the interlaminar shear strength is improved by adding a thin film containing short carbon fibers and carbon nanotubes at the overmolding interface. The optimization of the dispersion and forming conditions of carbon nanotubes significantly improved the interlaminar shear strength. To sum up, the interface treatment is the critical factor affecting the interfacial bonding strength in the overmolding process. Controlling the mold and melting temperatures, surface treatment, and other methods can improve the interfacial bonding strength.

However, regarding thermoplastic composites, reaching the melting temperature at the resin interface above (close to 340 °C) in the overmolding process is complex. In addition, it will become more difficult to remove the mold of the composite materials at high temperatures. Therefore, this paper uses PAEK as the resin matrix of continuous-fiber-reinforced composite materials with a relatively low melting temperature. With a high melting temperature, PEEK was applied as the resin matrix of the composite materials used in injection molding to prepare the overmolding materials. The melting temperature of the injection resin is expected to increase the interface temperature between the two materials to promote the mutual diffusion and fusion of resins at the interface and improve the interfacial bonding strength. This paper systematically studied the influence of mold and melting temperatures on composite materials’ interfacial bonding strength. The mechanical properties and morphology of the composites were characterized using short-beam shear and SEM, and the experimental results were verified with a molecular dynamics simulation.

## 2. Experimental Materials and Methods

### 2.1. Experimental Materials

This study prepared the continuous-fiber thermoplastic composite (CCF-PAEK) using a one-way prepreg (T700/PAEK, Yingchuang New Materials Co., Ltd., Heilongjiang, China). The fiber surface density was 146 g∙m^−2^, the fiber volume fraction was 63 vol%, the resin content was 30 wt.%, and the nominal thickness was 0.165 mm. The reinforced fiber was T700 carbon fiber produced by Tuozhan Carbon Fiber Co., Ltd., Weihai, China. The matrix resin was PAEK resin (PAEK-H, Hairuite Engineering Plastics Co., Ltd., Heilongjiang, China).

The injection molding materials were PEEK and short carbon-fiber-reinforced poly (ether ether ketone) (SCF-PEEK) (Heilongjiang Yingchuang New Materials Co., Ltd., Heilongjiang, China), with a short carbon fiber content of 30 vol%.

The thermal properties of PAEK and PEEK are shown in Table 1. The glass transition temperatures of PAEK and PEEK were 148 °C and 143 °C. The melting temperatures of PAEK and PEEK were 322 °C and 343 °C, respectively.

### 2.2. Preparation of the Composite Materials for Overmolding

**Figure 1:**
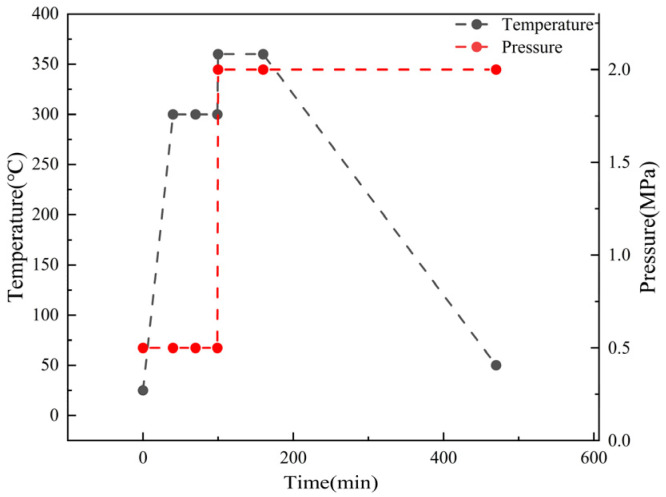
CCF-PAEK molding process.

Continuous-fiber-reinforced composite laminates were prepared through compression molding. First, the prepreg was cut according to the size of the mold cavity, and it was ultrasonically welded to prepare the prepreg (KH-2870Z, Kehai Automation Equipment Co, Weihai, China). Then, the preformed body was put into the mold cavity coated with a high-temperature release agent and put into the press (LSVI-50T, POTOP, Guangzhou, China) for molding. First, the mold was heated to 300 °C, the pressure was set to 0.5 MPa and maintained for 30 min. Then, the temperature was raised to 360 °C, the pressure was set at 2 MPa, and it was maintained for 60 min. Then, air cooling was applied, and the pressure was maintained until the temperature dropped below 140 °C. The molding process is shown in Figure 1. Because the research on continuous-carbon-fiber-reinforced composites is relatively mature, we chose a better method for preparing continuous-carbon-fiber-reinforced composites studied by other group members [22]. We only reviewed the injection molding process, which is more influential in overmolding. The lay-up modes of continuous-fiber-reinforced composites are as follows: the shear sample of the short beam is (0°)_16s_. The final preparation of CCF-PAEK fiber volume fraction was about 63 vol%, calculated as shown in Equation (2).

**Figure 2:**
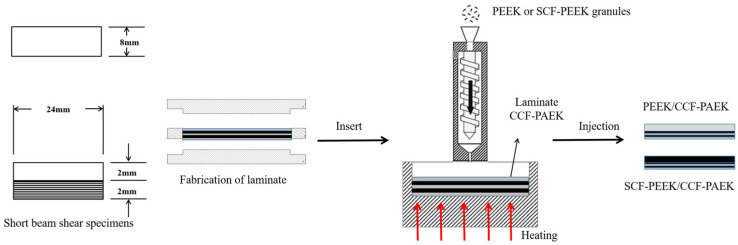
Sample size and overmolding process.

For the continuous-fiber-reinforced composite laminates obtained by molding, inserts were prepared with a water cutting machine (Proto MAX, OMAX, Kent, WA, USA), as shown in Figure 2. The inserts were put into an ultrasonic cleaning machine containing acetone solution (JP-100, Jie Ming, Shenzhen, China) for 30 min to remove residual release agent on the surface. After cleaning, the inserts were put in the oven at 100 °C to dry for 30 min (DZF-6020, Jinghong, Shanghai, China). Then, the dried inserts were placed into the mold cavity of the injection molding machine for preheating. The preheating time was set to 3 min so that the prefabricated part can reach a temperature close to the mold (TY-600, TAYU, Hangzhou, China). Where the preheating time is set to 3 min, the relationship between the temperature of the prefabricated parts and time is indicated in Figure S2. PEEK or SCF/PEEK was injected into the surface of continuous-fiber-reinforced composite laminates, and the pressure was maintained for 3 min. As shown in Table 2, single-factor experiments were designed to investigate the variation in shear strength of overmolding composites at different mold and melt temperatures, where A was used as the reference.

The technological parameters of overmolding are shown in Table 3. The higher melt temperature is conducive to transferring melt heat to the surface of the prefabricated parts prepared using PAEK at low melting temperatures so that the injection resin and the prefabricated parts form a good interface.

### 2.3. Test and Characterization

#### 2.3.1. Mechanics Performance Test

According to ASTM D2344 [23], a short-beam shear-strength test was performed. The thickness:width:span:length ratio of the sample was 1:2:4:6, the overmolding sample size was 4 mm × 8 mm × 24 mm, and the span was 16 mm. Each group was averaged according to the 5 experimental data in the standard. Then, the shear strength of the short beam was calculated according to Equation (1):

(1) $$F^{sbs}=0.75\times \frac{P_{m}}{b\times h}$$

where *F^sbs^* is the shear strength of the short beam; *P*_m_ is the maximum load when the material is damaged; *b* is the sample width; and *h* is the sample thickness.

#### 2.3.2. Morphology Analysis

A desktop scanning electron microscope (Regulus 8230, Hitachi, Tokyo, Japan) was used to characterize the micromorphology of the damaged section in the composite (the accelerating voltage was 5 kV). The surface of the damaged sample was pretreated with gold (spraying time of 60 s).

After using E54 epoxy resin and curing agent N,N-dimethylacetamide in a 10:1 mass ratio, the prepared resin solution was left for 30 min. The cut sample was fixed in a silicone mold (φ32 × h27 mm). The prepared resin was poured and cured in the electric blast-drying oven. Then, a gradient temperature rise was applied to the resin. After solidification, the metallographic sample was obtained and polished using sandpaper from small to large mesh numbers. Finally, nanopolishing liquid and a polishing cloth were selected for further polishing. Subsequently, a polarizing microscope (DM4-P, Leica, Germany) was used to observe the micromorphology of the samples.

#### 2.3.3. Fiber Volume Fraction of Composite Materials

The fiber volume fraction in continuous-carbon-fiber-reinforced composites prepared by molding is calculated according to Equation (1).

(2) $$\left(\rho_{1}/\rho_{2}\right)\times n/d=v$$

where *ρ*_1_ is the surface density of the prepreg; *ρ*_2_ is the bulk density of the fibers; n is the number of layups; d is the thickness of the prepreg layup; and v is the volume fraction of the continuous-carbon-fiber-reinforced composite.

#### 2.3.4. Rheological Behavior Analysis

The viscosity curves of PAEK and PEEK resin under a shear flow field were measured with a capillary rheometer (RG25, Gautford, Germany).

#### 2.3.5. Nanoindentation Test

The specimens were indented using a nanoindenter (G200X, KLA, Milpitas, CA, USA) to measure the microhardness and modulus at different locations. The advanced dynamic I and H methods were chosen for the tests, and the load was 25 mN.

#### 2.3.6. Molecular Dynamics Simulation

Molecular dynamics simulations were performed to reveal the bonding mechanism of PEEK and PAEK during overmolding (Material Studio 2020), and diffusion coefficients and binding energies at different interface temperatures were calculated. The diffusion coefficient *D* is determined according to Einstein’s relation [24,25] as follows:

(3) $$MSD=t{\langle |r_{i}\left(t\right)-r_{i}\left(0\right)|}^{2}\rangle$$

(4) $$D=\underset{t\to \infty }{\lim}\frac{1}{t}{\langle |r_{i}\left(t\right)-r_{i}\left(0\right)|}^{2}\rangle$$

where *r_i_* and *t* are the reference position and simulation time of each atom, and *r_i_* is the distance from the bit to the center of the mass of a single chain.

In the overmolding process, the formation of the interface depends not only on the mutual motion of the two-phase molecules but also on their self-motion. The radius of rotation (*R_g_*) of polymer molecules was studied to investigate the mechanism of molecular self-motion further. The movement depends on the size of the polymer macromolecules and their center of mass, calculated as follows:

(5) $$R_{g}=\sqrt{\frac{1}{M}}\underset{i}{\sum }m_{i}{\left(r_{i}-r_{cm}\right)}^{2}$$

where *M* and *r_cm_* are the total mass of the polymer chain in the system and the centroid position of the chain. It is assumed that multiple units are within the polymer chain. The mass of each element is mi, and *r_i_* is the distance from the atom to the centroid position of the individual chain. Usually, the rotation radius corresponds to the curl and contraction of the molecular chain. MSD and *R_g_* radius characterize the mobility of polymer molecules and chains.

The interface interaction energy between polymer layers mainly comprises van der Waals and electrostatic interaction energy. The interfacial bonding energy [26] is calculated as follows:

(6) $$E_{joining}=E_{total}-\left(E_{PAEK}+E_{PEEK}\right)$$

where *E_total_*, *E_PAEK_*, and *E_PEEK_* are the total energy of the system, the energy of the PAEK resin layer, and the energy of the PEEK resin layer, respectively.

An atomic model of the PEEK/CCF-PAEK interface was established to study the effect of mold temperature on the adhesion behavior of the interface during PEEK/CCF-PAEK overmolding. Because the resin matrix is located in the outer layer of the interface, an atomic model was adopted at the PEEK/PAEK interface. It was assumed that the bonding occurred during injection, without considering subsequent pressure preservation and cooling processes. The atomic models of PEEK and PAEK layers were also constructed. Some modeling parameters, including polymerization, number of chains, initial density, and size, are given in Table 4.

All calculations in this paper were performed using molecular dynamics simulations via VASP. The condensed phase-optimized molecular-potential force field describes the atomic interactions in atomic simulation studies (universal—a purely diagonal harmonic force field). The bond stretching is described in harmonic terms. Three Fourier cosine expansions define angular bending. Cosine Fourier expansions describe the torsion. The van der Waals interaction is described by the Leonard–Jones potential. Electrostatic interactions were described with atomic monopole and shielding (distance-dependent) coulomb terms. The aim is to achieve high precision in predicting the properties of very complex mixtures. All simulations were performed in a constant temperature–constant volume canonical ensemble (NPT). The Verlet algorithm was used to integrate the equations of motion. The integration time step was 1 fs, and the Nosé–Hoover thermostat controlled the temperature. The simulation system balances 300 ps to stabilize the interaction. After this phase, the total intermolecular interaction energy of 300 ps was recorded at 5 ps intervals. Finally, the average values were calculated to eliminate fluctuations in the simulation.

## 3. Results and Discussions

### 3.1. Influence of Mold Temperature on the Interfacial Bonding Strength

**Figure 3:**
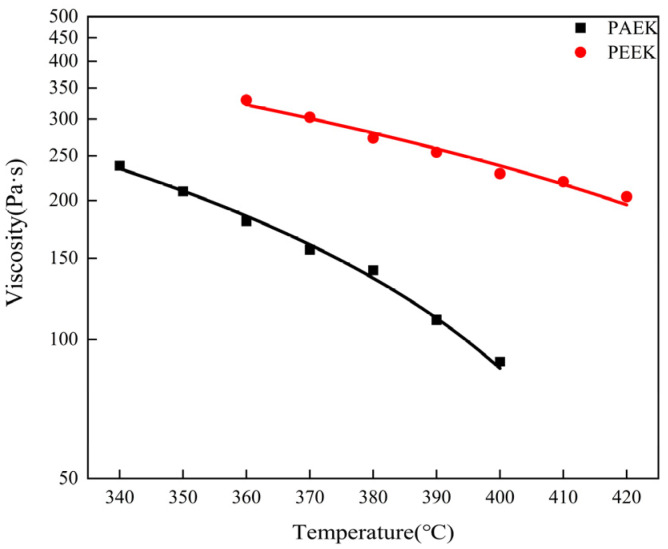
Temperature–viscosity curves of PAEK and PEEK resins.

The viscosity–temperature curves of PAEK and PEEK resin at the shear rate of 1000 s^−1^ are shown in Figure 3. The viscosity of PAEK at 340 °C~400 °C is about 89~237 Pa·s, and for PEEK at 360 °C~420 °C is 203~330 Pa·s. Both resins have shear-thinning behavior, and the viscosity changes tend to be consistent with the increase in temperature (the viscosity decreases with the increase in temperature). The lower the viscosity of the melt, the better the diffusion because the mixing process requires the melt to spread as far as possible at the interface before cooling.

**Figure 4:**
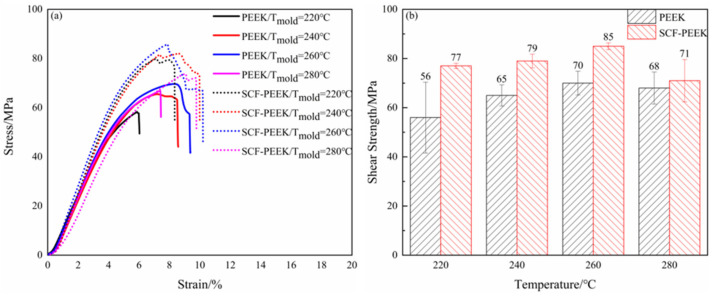
Influence of mold temperature on short-beam shear strength: (**a**) stress–strain curves of overmolding samples at different mold temperatures; (**b**) mold temperature and shear strength of the short beam.

The shear strengths of PEEK/CCF-PAEK and SCF-PEEK/CCF-PAEK at different mold temperatures are shown in Figure 4. In Figure 4a, the stress–strain curves of the two materials under different mold temperatures are shown. The strain of the sample increases slowly before it reaches the failure stress, and finally, shear failure occurs. The shear strength of the composite can be improved by adding short carbon fiber.

Figure 4b shows the shear strength of PEEK/CCF-PAEK and SCF-PEEK/CCF-PAEK short beams at different die temperatures. The shear strength of PEEK/CCF-PAEK is 56 MPa, 65 MPa, 70 MPa, and 68 MPa, respectively, whereas the shear strength of SCF-PEEK/CCF-PAEK is 77 MPa, 79 MPa, 85 MPa, and 71 MPa, respectively. The results show that the shear strength of the short beam can be improved with the increase in mold temperature. Mold temperature affects the interfacial temperature holding time between injection melt and insert and the contact time before curing. Therefore, with the increase in mold temperature, the temperature of the interface layer is gradually increased to promote the melting and diffusion of PAEK resin at low melting temperature and improve the interface bonding strength.

**Figure 5:**
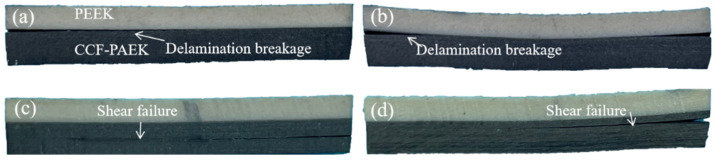
PEEK/CCF-PAEK, composite failure samples, prepared at different mold temperatures: (**a**) 220 °C; (**b**) 240 °C; (**c**) 260 °C; and (**d**) 280 °C.

**Figure 6:**
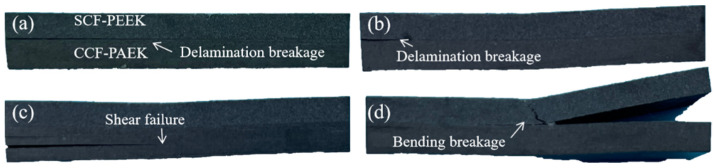
SCF-PEEK/CCF-PAEK, composite failure samples, prepared at different mold temperatures: (**a**) 220 °C; (**b**) 240 °C; (**c**) 260 °C; and (**d**) 280 °C.

Shear failure modes of short beams of overmolding composites at different mold temperatures are shown in Figure 5 and Figure 6. Under shear force, cracks begin to occur on both sides of the sample and expand to the middle. The failure of PEEK/CCF-PAEK was mainly caused by the interface delamination when the mold temperatures were 220 °C and 240 °C. In this case, the interfacial bonding strength was weak. PEEK/CCF-PAEK failure was mainly caused by the interlayer fracture when the mold temperatures were increased to 260 °C and 280 °C (Figure 5c,d). In this case, a strong interfacial bonding was observed.

Similarly, the failure of SCF-PEEK/CCF-PAEK was similar to that shown in Figure 6. The interfacial bonding failure was the main issue when the mold temperatures were 220 °C and 240 °C. In the latter case, the cracks at the interface gradually became smaller. When the mold temperatures increased to 260 °C and 280 °C; the failure of SCF-PEEK/CCF-PAEK was an interlaminar fracture of CCF-PAEK and bending failure of SCF-PEEK. Due to the flexural deformation and interlaminar shear deformation by overmolding, the lamination between PEEK, SCF-PEEK, and CCF-PAEK will occur when the interfacial bonding strength weakens. With the increase in interfacial bonding strength, the interfacial delamination of the composite decreases gradually, and the interlaminar fracture of the resin increases. Wang et al. [27] studied the effect of resin content in polyimide/bisphenol A diisocyanate laminates on the shear strength of short beams. They showed that a lower resin content would limit the full infiltration of the fibers and reduce the shear strength of short composite beams. In overmolding composites, the resin content of the PEEK or SCF-PEEK side is more than that of CCF-PAEK. It should be noted that PEEK resin has a higher toughness. Therefore, when the interfacial bonding strength of overmolding composite materials is higher, shear delamination failure no longer occurs at the interface but occurs inside the laminate.

**Figure 7:**
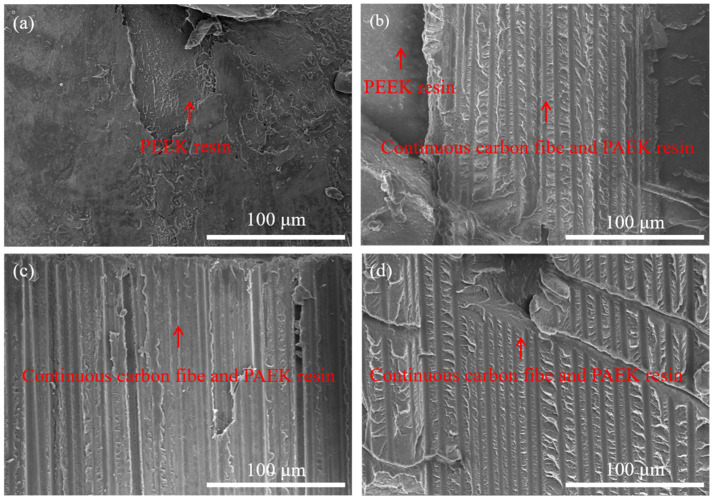
Failure morphology of PEEK surface at different mold temperatures: (**a**) 220 °C; (**b**) 240 °C; (**c**) 260 °C; and (**d**) 280 °C.

We observed the specimen failure mode and the microscopic morphology of the resin surface after specimen destruction, where the direction of the destruction morphology observation is indicated in Figure S3. At a mold temperature of 220 °C, i.e., Figure 7a, the PEEK damage surface morphology is relatively smooth, and only the PEEK resin portion can be observed. When the mold temperature is raised to 240 °C, i.e., Figure 7b, the PEEK resin surface is adhered to the CCF-PAEK portion of the continuous carbon fiber and its resin, indicating that resin melting begins to occur at the PEEK/CCF-PAEK interface at this time [28]. However, at this time, the interfacial bond strength is relatively low, and shear damage still occurs at the interface, as shown in Figure 5b. Until the mold temperature is raised above 260 °C as shown in Figure 7c,d, only continuous fibers and their resin in CCF-PAEK can be observed in the damage morphology at this time, and combined with Figure 5c,d, it can be seen that shear damage occurs at the interlayer of CCF-PAEK at this time.

**Figure 8:**
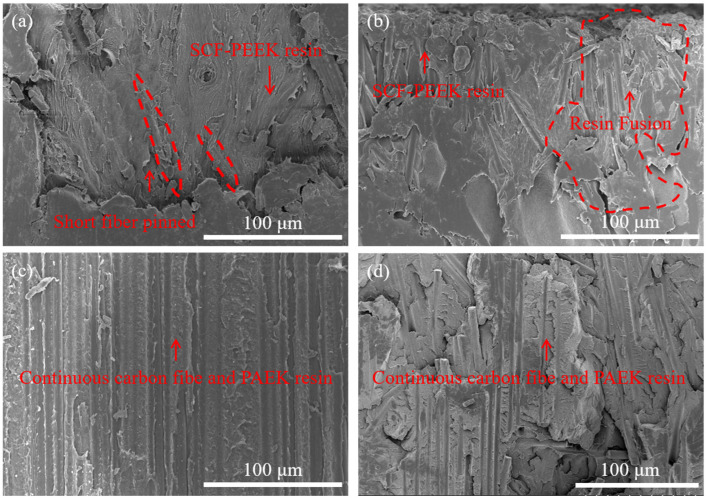
Failure morphology of SCF-PEEK surface at different mold temperatures: (**a**) 220 °C; (**b**) 240 °C; (**c**) 260 °C; and (**d**) 280 °C.

The microscopic morphology corresponding to the shear damage of SCF-PEEK/CCF-PAEK in Figure 8 is the same as that of PEEK/CCF-PAEK, and only SCF-PEEK can be observed at a mold temperature of 220 °C. As the mold temperature increases, as shown in Figure 8b, the appearance of continuous carbon fibers in the red circle indicates that melting begins to occur at the interface. As the mold temperature increases to 260 °C, i.e., Figure 8c, only continuous fibers of CCF-PAEK and its resin can be observed at the interface.

The experimental results show that the interface failure mode of the composite changes with the increase in mold temperature. When it is low, the temperature at the interface is low, the injection mold melt cools rapidly, and the molecular diffusion is relatively slow, resulting in poor adhesion [29]. Shear failure is manifested as an interface failure. Interfacial bonding is manifested as mechanical bonding. With the increase in the mold temperature, the fracture surface area of PEEK increased gradually. Higher mold temperature increased the interface temperature between PEEK resin and PAEK and increased the blending time before curing, which was beneficial to the fusing process of the resin. When the interface temperature is higher than the melting temperature of PAEK, the resin eutectic layer is formed at the interface [30], which improves the interfacial bonding strength.

**Figure 9:**
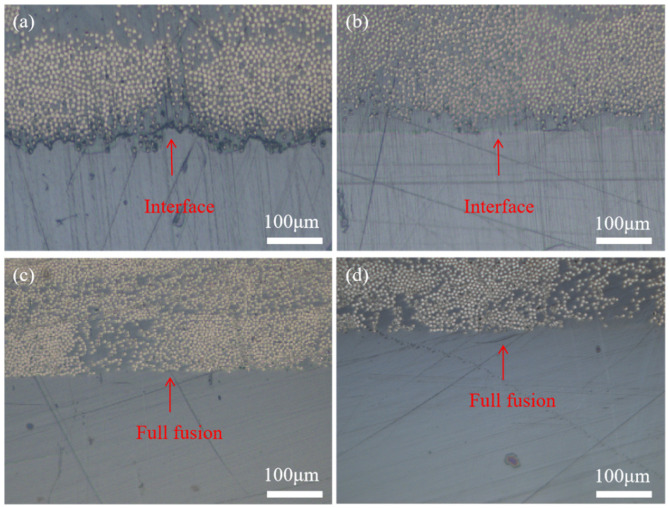
The interface of PEEK/CCF-PAEK before failure at different mold temperatures: (**a**) 220 °C; (**b**) 240 °C; (**c**) 260 °C; and (**d**) 280 °C.

**Figure 10:**
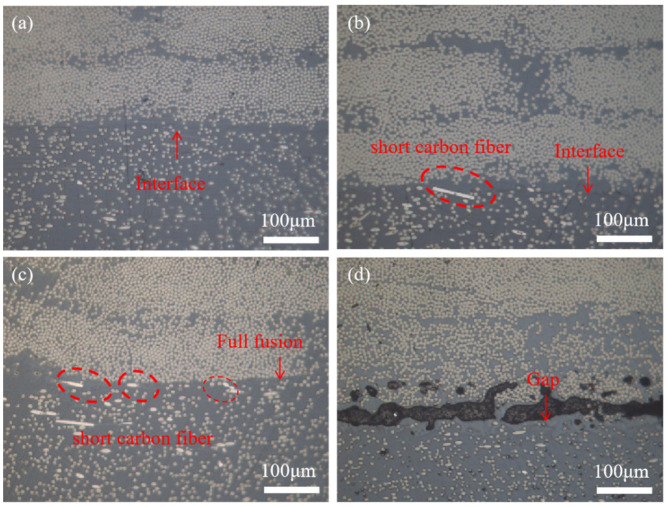
The interface of SCF-PEEK/CCF-PAEK before failure at different mold temperatures: (**a**) 220 °C; (**b**) 240 °C; (**c**) 260 °C; and (**d**) 280 °C.

The interfaces of PEEK/CCF-PAEK and SCF-PEEK/CCF-PAEK before failure are shown in Figure 9 and Figure 10, respectively. Optical microscope observations were made at different mold temperatures, with CCF-PAEK on the upper side and PEEK and SCF-PEEK on the lower side. In Figure 9a, when the mold temperature was 220 °C, PEEK resin was poorly combined with PAEK resin, resulting in obvious interface stratification and even pores. When the mold temperature is 220 °C, there is also a boundary at the interface, but no pores are observed (Figure 10a). With the increase in mold temperature, the boundary line slowly disappears. Although there was no obvious gap between PAEK and PEEK, a clear boundary could still be observed (Figure 9a,b and Figure 10a,b). The interface between the two completely disappears when the mold temperature reaches 260 °C. The results show that resin melting can significantly improve the shear strength of short beams, which is of great significance. After adding the short carbon fiber, they cross the boundary and are nailed in the PAEK resin layer (Figure 10b,c).

Adding staple fibers enhances the stiffness and strength of the injection layer but also influences the interfacial bonding strength [31]. However, when the mold temperature was raised to 280 °C, holes appeared at the interface (Figure 10d). In the process of overmolding, when the mold temperature is 280 °C, the resin adheres to the mold cavity, and the difficulty of demolding leads to defects at the interface layer, which reduces the interlayer shear strength.

The results show that the interface between the injection and insert layers will change with different mold temperatures. With the increase in mold temperature, the melt can have a longer contact time before consolidation. Thus, changing the melting state of PAEK resin at low melting temperatures promotes the heat transfer between the injection molding layer and the insert layer, making the PAEK and PEEK interface disappear while forming the resin eutectic layer. The boundary line between the injection mold melt and the insert interface gradually disappears, forming a strong and reliable connection and increasing the shear strength. The mold temperature is still lower than the melting temperature of PAEK. Still, the heat transferred by PEEK at high melting temperatures combines the injection and insert layers. It can solve the problem that the composite material of overmolding cannot be molded at high temperatures.

**Figure 11:**
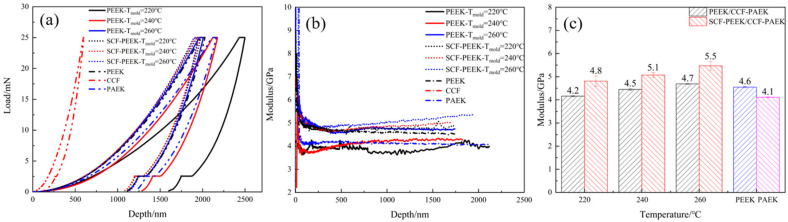
Nanoindentation analysis of composite materials formed by secondary molding at different mold temperatures: (**a**) load depth curve; (**b**) modulus depth curve; (**c**) modulus.

The nanoindentation load-depth curves of the secondary molded composites at different mold temperatures are shown in Figure 11a. Observing the curves in the Figure, we know that for the same indentation load, the indentation depth gradually becomes smaller as the mold temperature increases, indicating that the bearing capacity of the resin at the interface gradually becomes stronger as the mold temperature increases. For the PEEK/CCF-PAEK composite, the load capacity of the interface resin is similar to that of PEEK when the mold temperature is 260 °C, indicating that the preform and the injection layer resin have reached a state of molten resin intermixing, which is consistent with the strength of PEEK. The higher loads at the SCF-PEEK/CCF-PAEK composite interface compared to PEEK indicate that adding short-cut carbon fibers enhances the resin at the interface, allowing it to carry higher loads.

It can be observed that the modulus decreases rapidly with the increase in the indentation depth when the indentation depth is small in Figure 11b, and the modulus curve changes more at this time. After the depth exceeds 250 nm, the modulus value gradually becomes smooth with the increase in depth. When the indentation depth exceeds 500 nm, the modulus curve becomes smooth, and the material modulus is calculated at this time. The depth-modulus curve of the PEEK/CCF-PAEK secondary composite at a mold temperature of 220 °C is relatively unstable, and the modulus at this time is low at 4.2 GPa. This indicates that the melt can form a resin intergradation layer with the preform surface resin at a mold temperature of 260 °C and, therefore, has the same modulus as PEEK. For the SCF-PEEK/CCF-PAEK secondary molding composite, the depth–modulus curve is relatively smoother, and the addition of short-cut carbon fibers can improve the modulus of the resin at the interface. With the increase in mold temperature, the modulus also gradually increased. The increase was higher at the mold temperature of 260 °C. In the previous paper, the modulus reached 5.5 GPa, related to transforming the interfacial bonding state at the mold temperature of 260 °C, i.e., the interfacial resin can melt and diffuse into one. The short-cut carbon fiber can be embedded in the interfacial layer, which benefits the modulus increase.

### 3.2. Influence of Melt Temperature on the Interfacial Bonding Strength

**Figure 12:**
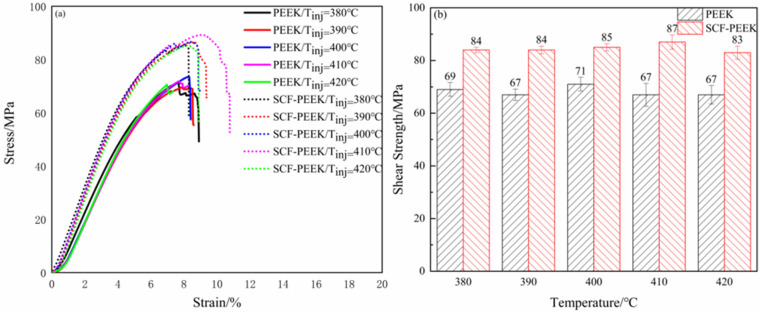
Influence of melt temperature on short-beam shear strength: (**a**) stress–strain curves of overmolding samples at different melt temperatures; (**b**) melt temperature and shear strength of the short beam.

**Figure 13:**
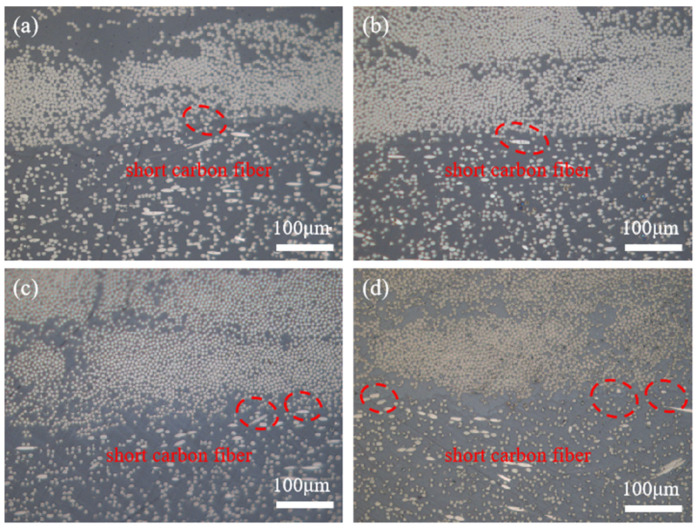
The interface of SCF-PEEK/CCF-PAEK before failure at different melt temperatures: (**a**) 380 °C; (**b**) 390 °C; (**c**) 410 °C; and (**d**) 420 °C.

Figure 12 shows the shear strength of PEEK/CCF-PAEK and SCF-PEEK/CCF-PAEK short beams at different melting temperatures. The shear strength of PEEK/CCF-PAEK was 69 MPa, 67 MPa, 71 MPa, 67 MPa, and 66 MPa, respectively. The shear strength of the SCF-PEEK/CCF-PAEK short beam was 84 MPa, 84 MPa, 85 MPa, 87 MPa, and 83 MPa, respectively. Comparing the shear strength data of the short beam of the two resin overmolding samples shows that the melt temperature has little influence on the PEEK/CCF-PAEK interfacial bonding strength when the mold temperature is 260 °C. Figure 13 shows the interfacial bonding state of the SCF-PEEK/CCF-PAEK composite at different melt temperatures, and the boundary between PAEK and PEEK becomes unclear when the mold temperature is 260 °C. As the melt temperature increases, more and more short carbon fibers are pinned into the resin; as shown in the red circle in the figure, the short carbon fibers cross the boundary to connect the two matrix resins and improve the interfacial bonding strength. The fluidity of the SCF-PEEK resin can be improved by increasing the melt temperature when a resin interfusion zone forms at the interface. More short carbon fibers can be inserted into the resin-rich region to strengthen the interface.

The final conditions chosen were a mold temperature of 260 °C, the highest shear strength at a melt temperature of 400 °C for PEEK/CCF-PAEK, and a higher melt temperature of 410 °C for SCF-PEEK/CCF-PAEK. According to Figure S1. For the relationship between interface temperature and shear strength for PEEK/CCF-PAEK, below the interface temperature of 320 °C, the shear strength of the melt is lower than at the interface temperature of 320 °C. When the interface temperature is raised to 320 °C and above, the shear strength at low mold temperature and high melt temperature is lower than that at high mold temperature. At high mold temperatures, the melt temperature has less influence on the shear strength.

### 3.3. Molecular Dynamics Simulation

**Figure 14:**
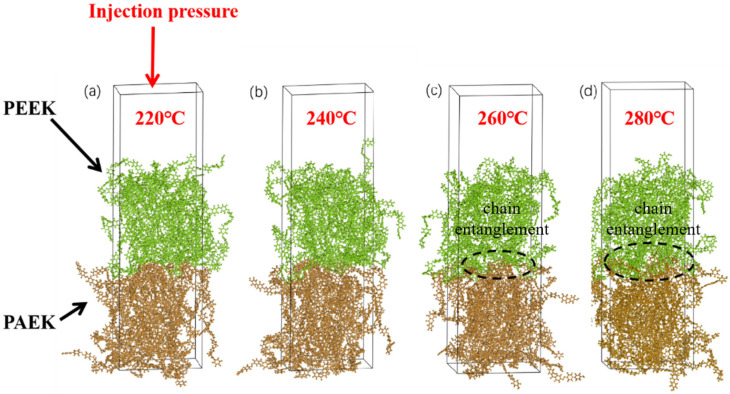
Adhesion of PEEK and PAEK interfaces at different mold temperatures: (**a**) 220 °C; (**b**) 240 °C; (**c**) 260 °C; and (**d**) 280 °C.

PAEK was painted brown to observe the intermolecular diffusion and interface formation process, and PEEK was painted green, as shown in Figure 14. According to the results, the mold temperature significantly affects the interfacial bonding strength. In contrast, melt temperature has nearly no effect. Therefore, this simulation used only mold temperature as the influencing factor. The injection molding temperature is set at 400 °C, and the mold temperature is set at 220 °C, 240 °C, 260 °C, and 280 °C, respectively. The simulation showed that as the mold temperature increased, some molecular chains crossed the interface and became entangled with another layer of molecular chains.

**Figure 15:**
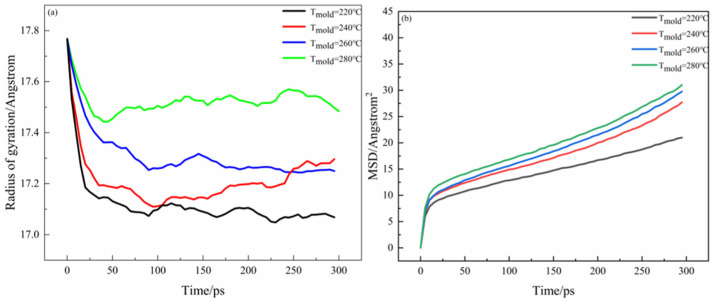
R_g_ and MSD at PEEK and PAEK interfaces: (**a**) rotation of gyration; (**b**) mean square displacement.

Figure 15 shows the radius of gyration, mean square displacement of the whole system during the interface bonding process at different mold temperatures. In PEEK/PAEK overmolding, the formation of the interface depends not only on the mutual motion of the two molecular chains but also on the self-motion of the molecules. Figure 14 shows the rotation radius and mean azimuth shift data at different mold temperatures at the PAEK and PEEK interfaces. It can be seen that the rotation radius of PEEK and PAEK increases with the increase in mold temperature as can be seen in Figure 14a that under different processing conditions, the rotation radius of the total system gradually increases when it reaches a stable state at 300 ps. Figure 14b shows the mean azimuth shift–time curve at different mold temperatures. At higher values, the total mean azimuth shift increases rapidly with time. This means that as the temperature increases, the molecules move faster. However, when the temperature change is the same, especially when the mold temperature is 220 °C, the change in MSD is slower. It was found that under the processing conditions, the mechanical properties of the prepared samples and the microstructure observation after failure showed slow molecular movement and poor interfacial bonding strength. The molecular motion rate increases gradually with the increase in mold temperature, and the interface bonding strength increases. However, the mean azimuth shift value is small when the mold temperature gradually increases to 260 °C and 280 °C. The experimental results show that the interfacial bonding strength does not increase after the mold temperature increases.

**Figure 16:**
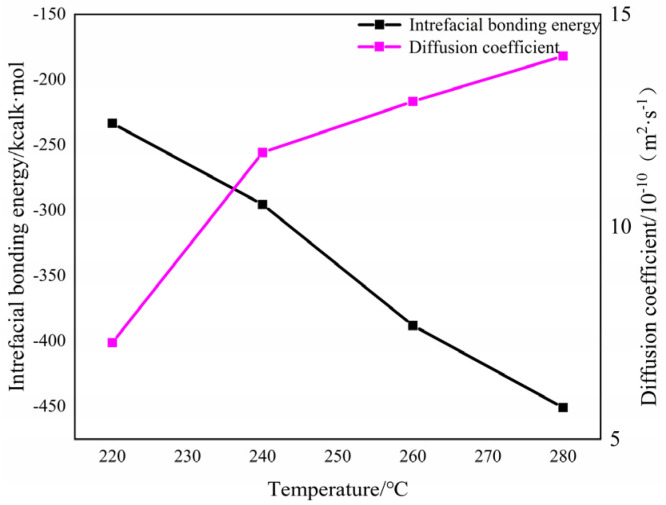
The two systems interfacial bonding energy and diffusion coefficient at different mold temperatures.

Figure 16 shows the two systems’ interface bonding energy and diffusion coefficient at different mold temperatures. It can be seen that when the mold temperature increases from 220 °C to 280 °C, the diffusion coefficient increases from 7.3 × 10^−10^ m^2^∙s^−1^ to 14.0 × 10^−10^ m^2^∙s^−1^, and the absolute value of the interface energy increases sharply from 233.4 kcal∙mol^−1^ to 450.8 kcal∙mol^−1^. Compared with other temperature changes, the diffusion coefficient changes are larger when the mold temperature increases from 220 °C to 240 °C. At this time, the molecular diffusion rate increased, which was in the same trend as the shear strength of the short beam, as shown in Table 5. It is proved that the resin began to melt and bond at the interface within the mold temperature range from 220 °C to 240 °C. It can be concluded that the non-bonding free energy at the interface increases when the mold temperature increases, and the mutual diffusion of the two systems at the interface is enhanced, thus improving the interface energy.

In the molecular dynamics simulation, the interfacial bonding energy and diffusion coefficient will continue to increase when the mold temperature increases. Only the injection process is calculated without considering processes such as holding pressure, and only the resin molecular chain is simulated without involving fibers. The simulation results do not match the experimental results at the final mold temperature of 280 °C, as the interfacial bonding energy is higher in the simulation. Still, the actual interfacial bonding strength is highest at the mold temperature of 260 °C due to the internal stress at the interface of the overmolding composite at the high mold temperature, so the final mold temperature is 260 °C.

## 4. Conclusions

(1) In this paper, carbon-fiber-reinforced composite material (CCF-PAEK) with a low melting temperatures was prepared via PAEK. PEEK and SCF-PEEK resin with high melting temperatures were used as injection resin. The results show that the interface bonding strength is greatly affected by mold temperature, whereas the melting temperature has little effect on the interface bonding strength. When the mold temperature was 220 °C, the shear strength of the PEEK/CCF-PAEK short beam was 56 MPa, and when the mold temperature was increased to 260 °C, the shear strength of the PEEK/CCF-PAEK short beam was 70 MPa, which increased by 25%. The shear strength of short-beam SCF-PEEK/CCF-PAEK was 77 MPa when the mold temperature was 220 °C and 85 MPa when the mold temperature was raised to 260 °C. When the mold temperature was lower than the melt temperature of PAEK, the overmolding composites can form a good bond. The melt temperature had little effect on the shear strength of the PEEK/CCF-PAEK short beams, but as the melt temperature increased from 380 °C to 410 °C, the shear strength of the SCF-PEEK/CCF-PAEK short beams increased from 83 MPa to 87 MPa. For the SCF-PEEK/CCF-PAEK, the increase in melt temperature can promote more short carbon fibers to cross the boundary and nail more short carbon fibers in the matrix interfusion zone to improve the shear strength of short beams.

(2) The interfacial bonding of overmolding composites includes mechanical meshing, resin fusion, and short carbon fiber interfacial nailing. The molecular dynamics simulation and experiments prove that the interface bonding strength mainly depends on mechanical meshing when the mold temperature is less than 220 °C. The resin melts at the interface when the mold temperature is 220–240 °C. Mechanical meshing and resin fusion affect the interface bonding strength in this case. When the mold temperature reaches 260 °C, the resin at the interface is completely fused, forming the matrix interfusion zone. The short carbon fiber can cross the interface and connect the two resin matrices, improving the bonding strength.

(3) Through the molecular dynamics simulation, it was found that the interface bonding energy and diffusion coefficient are in good agreement with the experimental results. The mold temperature had a positive effect on PAEK-PEEK interface bonding. The diffusion coefficient and bonding energy of the interface increased with the increase in mold temperature. Therefore, a higher mold temperature will result in higher interface bonding strength.

## Figures and Tables

**Table 1:** Thermal properties of PAEK and PEEK.

Resin	T_g_/°C	Melting/°C		
		T_onset_	T_peak_	T_final_
PAEK	148	309	322	330
PEEK	143	334	343	347

**Table 2:** Single-factor experimental design table.

Num	T_mold_/°C	T_inj_/°C	Polymer Component
A	260	400	PEEK & SCF-PEEK
1	220	380	
2	240	390	
3	280	410	
4	/	420	

**Table 3:** Overmolding process parameters.

Num	T_mold_/°C	T_inj_/°C	Polymer Component
1	220	400	PEEK & SCF-PEEK
2	240	400	
3	260	400	
4	280	400	
5	260	380	
6	260	390	
7	260	410	
8	260	420	

**Table 4:** The main parameters of the atomic model of PEEK and PAEK.

Material	Number of Chains	Degree of Polymerization	Total Amount of Atoms	Initial Density /g·cm^3^	Box Size /nm^3^
PEEK	17	5	5542	1.3	4 × 6.2 × 2.4
PAEK	10	10	5886	1.3	4 × 6.2 × 2.4

**Table 5:** Interfacial bonding energy, diffusion coefficient, and shear strength of PEEK and PAEK short beam.

T_mold_ /°C	Diffusion Coefficient /10^−10^m^2^·s^−1^	Bonding Energy /kcal·mol^−1^	PEEK/CCF-PAEK ILSS/MPa	SCF-PEEK/CCF-PAEK ILSS/MPa
220	7.2	233.3	56	77
240	11.8	295.4	65	79
260	13.0	388.0	70	83
280	14.0	450.8	68	71

## Data Availability

Not applicable.

## Supplementary Materials

The following supporting information can be downloaded at: https://www.mdpi.com/article/10.3390/ma16124456/s1, Figure S1. Relationship between interface temperature and shear strength. Figure S2. Surface temperature of prefabricated parts in relation to preheating time. Figure S3. Direction of damage morphological observation.
