# Supplementary material for: Study on the Overmolding Process of Carbon-Fiber-Reinforced Poly (Aryl Ether Ketone) (PAEK)/Poly (Ether Ether Ketone) (PEEK) Thermoplastic Composites
Source: Materials (Basel). 2023 Jun 18;16(12):4456. doi: 10.3390/ma16124456 (PMC10305048; doi:10.3390/ma16124456)
Supplement: Supplementary file 1 [file materials-16-04456-s001.zip › materials-2415474-supplementary.pdf]

Electronic Supplementary Information (ESI):

Study on the Overmolding Process of Carbon-Fiber-Reinforced Poly (Aryl Ether Ketone) (PAEK)/Poly (Ether Ether Ketone) (PEEK) Thermoplastic Composites

Ziyue Zhao, Jindong Zhang, Ran Bi, Chunhai Chen, Jianan Yao * and Gang Liu *

Center for Advanced Low-Dimension Materials, State Key Laboratory for Modification of Chemical Fibers and Polymer Materials, College of Materials Science and Engineering, Donghua University,
Shanghai 201620, China
* Correspondence: yjn@dhu.edu.cn (J.Y.); liugang@dhu.edu.cn (G.L.);
Tel.: +86-21-67798670 (J.Y.)

List of Contents for Supplementary Materials:

Figure S1. Relationship between interface temperature and shear strength

Figure S2. Surface temperature of prefabricated parts in relation to preheating time

Figure S3. Direction of damage morphological observation

According to the method used in [1] to calculate the interface temperature, i.e:

$$T_i = (T_{mold} + T_{inj})/2 \quad (1)$$

Where T_i is the interface temperature, T_{mold} is the mold temperature and T_{inj} is the injection temperature. Interface temperature through the mold temperature and melt temperature together control, according to Figure 1 shows, shear strength in the interface temperature of 320 °C after the stabilization, thereafter continue to increase the interface temperature and the impact on the shear strength is smaller, Figure 1 can be learned in the interface temperature of 320 °C, the mold temperature of 260 °C when the shear strength is higher, the final choice of mold temperature 260 °C, melt temperature 400 °C.

[1] Jiang, B.; Fu, L.; Zhang, M.; Weng, C.; Zhai, Z. Effect of thermal gradient on interfacial behavior of hybrid fiber reinforced polypropylene composites fabricated by injection overmolding technique. *Polym. Compos.* **2020**, *41*, 4064–4073. https://doi.org/10.1002/pc.25693.

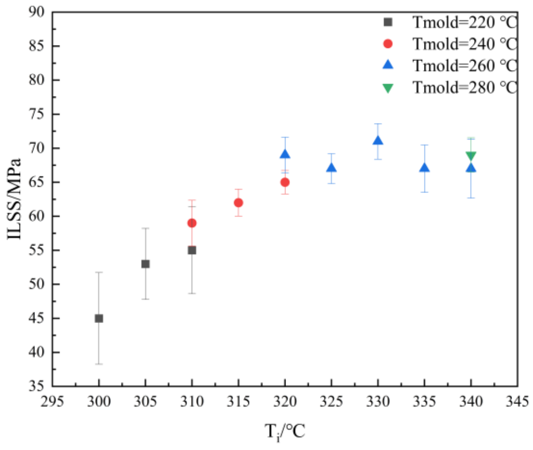

Figure S1. Relationship between interface temperature and shear strength

According to the relationship between the surface temperature of the precast and the preheating time, the surface temperature reaches 260 °C when the preheating time reaches 2 min, but in order to ensure that the whole precast reaches the preset temperature, the preheating time is set to 3 min. It can be observed that the surface temperature of the precast does not change from 3 min to 4 min, ensuring that the precast reaches the preset temperature. Therefore, a preheating time of 3 min was chosen.

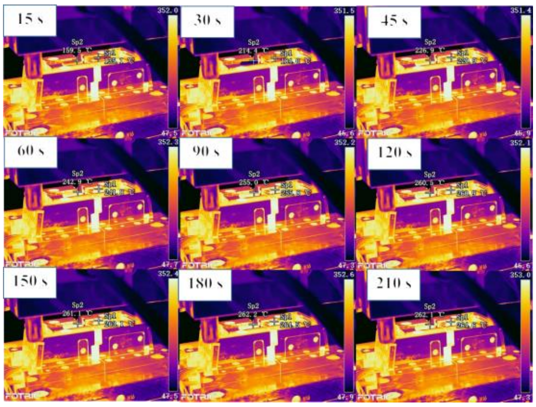

Figure S2. Surface temperature of prefabricated parts in relation to preheating time

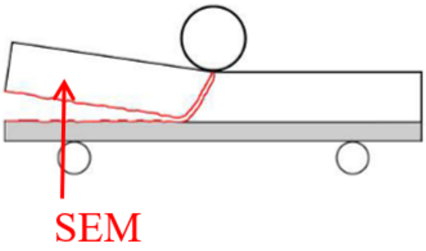

Figure S3. Direction of damage morphological observation
